# TTK promotes mesenchymal signaling via multiple mechanisms in triple negative breast cancer

**DOI:** 10.1038/s41389-018-0077-z

**Published:** 2018-09-12

**Authors:** Jamie L. King, Baotong Zhang, Yixiang Li, Kathy P. Li, Jianping J. Ni, Harold I. Saavedra, Jin-Tang Dong

**Affiliations:** 1 Winship Cancer Institute, Department of Hematology and Medical Oncology, Emory University School of Medicine, Atlanta, GA USA; 2 Cancer Biology Graduate Program, Emory University Laney Graduate School, Atlanta, GA USA; 3 Department of Pharmacology, Ponce Health Sciences University School of Medicine, Ponce Research Institute, Ponce, Puerto Rico

## Abstract

Abnormal expression of TTK kinase has been associated with the initiation, progression, and therapeutic resistance of breast and other cancers, but its roles remain to be clarified. In this study, we examined the role of TTK in triple negative breast cancer (TNBC), and found that higher TTK expression correlated with mesenchymal and proliferative phenotypes in TNBC cells. Pharmacologic inhibition and genomic silencing of TTK not only reversed the epithelial-to-mesenchymal transition (EMT) in TNBC cells, but also increased the expression of KLF5, an effector of TGF-β signaling and inhibitor of EMT. In addition, TTK inhibition decreased the expression of EMT-associated micro-RNA miR-21 but increased the expression of miR-200 family members and suppressed TGF-β signaling. To test if upregulation of KLF5 plays a role in TTK-induced EMT, TTK and KLF5 were silenced simultaneously, which reversed the decreased EMT caused by loss of TTK. Consistently, the decrease in miR-21 expression and increase in miR-200 expression caused by TTK silencing were rescued by loss of KLF5. Altogether, this study highlights a novel role and signaling pathway for TTK in regulating EMT of TN breast cancer cells through TGF-β and KLF5 signaling, highlighting targetable signaling pathways for TTK inhibitors in aggressive breast cancer.

## Introduction

Triple negative breast cancer (TNBC) remains a critical public health issue, is characterized by lack of progesterone, estrogen, and HER2 expression and is more aggressive than other breast cancer subtypes. As a result of lacking ER, PR, and HER2, which are well-established pharmaceutical targets for breast cancer treatments, targeted treatments against TNBC have yet to be developed. Also, while basal (76% of TNBC) and Her2 + breast cancers respond better to chemotherapy than luminal subtypes, they have a higher probability of relapse^1^. Thus, directed therapies against TNBC would improve survival outcomes of these patients.

Molecular profiling within TNBC revealed genetic differences that could affect cellular behavior and responses to chemotherapy. TNBC subtypes include four groups (BL1, BL2, M, and LAR^2^). The mesenchymal subtype is enriched in genes associated with the epithelial-to-mesenchymal transition (EMT), which is associated with increased cell migration, invasion, tumor metastasis, and resistance to chemotherapy and radiotherapy^3,4^. Mesenchymal TNBC cells exhibit a loss of epithelial morphology, increased cell migration and invasion. EMT may contribute to TNBC progression because it is an early event during cancer metastasis. EMT may also be mediated by changes in molecules that control mitosis and centrosome biology^5–7^, but mitotic proteins that regulate EMT phenotypes have not been fully characterized.

TNBC, Her2+, and ER− breast cancers have elevated frequencies of centrosome amplification (CA) and chromosome instability (CIN) relative to other subtypes^8,9^. We previously noted overexpression of the TTK kinase in Her2+ (ER-PR-) breast cancer cells displaying elevated CA compared to untransformed mammary epithelial cells^10^. CA causes mammary tumorigenesis in mice and correlates with high stage, grade, and poor relapse-free and overall survival of breast cancer patients^11,12^, which suggests a role for TTK in mammary tumor development and progression. CA also induces CIN, de-differentiation, and invasion, which suggest underlying mechanisms for TTK’s role in tumorigenesis and EMT^13,14^.

TTK plays critical roles in aneuploidy and genomic integrity across cancer types^15–21^, and is reported to function in promoting cell invasion^22^. In breast cancer, high TTK expression correlates with aggressive subtypes and therapeutic resistance^10,17,20,23,24^. Additionally, higher TTK expression was noted among a group of spindle assembly regulators in breast cancer cell lines and patient samples^19^. We found TNBC cell lines exhibited the highest levels of TTK, while knockdown of TTK increased apoptosis and prevented tumor growth^17,20^. Genomic silencing of TTK in Her2+ breast cancer cells attenuates CA^10^, as does pharmacological inhibition (unpublished data). Although TTK has higher expression in TNBC cells compared to other subtypes, distinct biological functions of TTK in TNBC remain unclear.

In this study, we hypothesized that TTK exerts functions in mesenchymal TNBC cells by regulating KLF5 and associated miR-21 or miR-200 microRNAs, since TTK functions in TGF-β signaling. We show the mesenchymal status of TNBC cells is decreased by targeting TTK, which is dependent on KLF5 upregulation. We also show silencing TTK reversed expression patterns of miR-21 and miR-200 family members, in parallel with the decreased mesenchymal phenotype. Our study highlights a distinct TTK-induced signaling pathway in TNBC where TTK maintains the proliferative and EMT phenotype by suppressing KLF5, which facilitates upregulation of miR-21 expression and downregulation of miR-200s. Clinical therapeutic strategies could be developed for TNBC by targeting this novel signaling pathway.

## Results

### Increased TTK expression is correlated with higher tumor grade, triple negative status, and worse overall survival in breast cancer

**Fig. 1:**
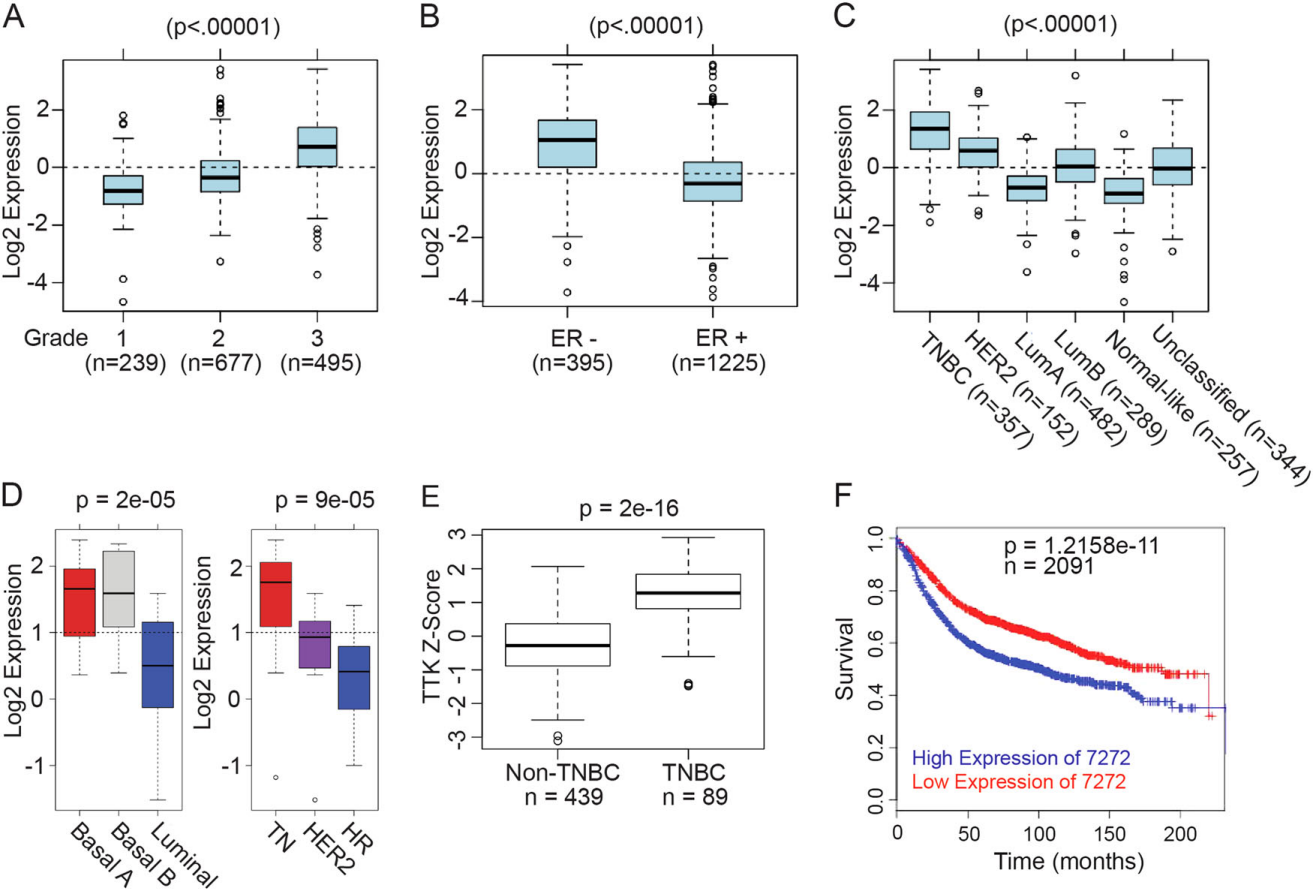
**Increase in TTK expression and its correlation with higher tumor grade, triple negative status, and worse overall survival in breast cancer**. **a**–**d** Box plots of TTK gene expression across tumor grades (**a**), between ER-positive and ER-negative breast cancers (**b**), across Pam50 subtypes of tumors (**c**), and across different subtypes in breast cancer cell lines from the GOBO database (**d**). **e** Box plots comparing TTK z-scores between TNBCs and non-TNBCs from patients in the TCGA database. **f** Kaplan–Meier survival analysis illustrating the correlation between higher TTK expression and decreased overall survival in breast cancer patients from the BreastMark database

To determine the significance of higher TTK expression in breast cancer, we analyzed TTK expression in three publically-available platforms. First, TTK expression was analyzed in the GOBO database^25^. Analyzing TTK expression across tumor grades indicated high TTK expression significantly correlated with grade III breast tumors compared to grades I or II tumors (Fig. 1a). TTK expression was also compared between ER-negative and ER-positive tumors, showing a significant correlation betwcompared to HER2+ and ER+ and/or PR+ celleen higher TTK expression and ER negativity (Fig. 1b). Further analysis of tumors from different clinical subtypes of breast cancer in the GOBO database revealed higher TTK expression was most-significantly correlated with triple negative (TN) subtypes compared to all other subtypes (Fig. 1c). Analysis of breast cancer cell lines in the GOBO samples showed higher TTK expression correlated with Basal A and Basal B subtypes compared to the luminal subtype (Fig. 1d). When categorized by clinical subtypes, higher TTK expression was correlated with the TN subtype compared to HER2+ and ER+ and/or PR+ cell lines (Fig. 1d). We then analyzed TTK expression in RNAseq data generated from breast cancers from TCGA. The level of TTK expression was significantly higher in the TNBC cases (*n* = 89) when compared to all non-TNBC cases (*n* = 439) (Fig. 1e). We next tested whether increased TTK expression correlates with patient survival using the BreastMark database^26^. Among 2091 breast cancer patients with both TTK expression and survival data available, higher TTK expression significantly correlated with decreased overall survival (Fig. 1f). These results indicate higher TTK expression correlates with aggressive features of breast cancer, including higher tumor grade, ER negativity, worsened survival, and TN status.

### TNBC cell lines are sensitive to anti-proliferative activity of TTK inhibitor

**Fig. 2:**
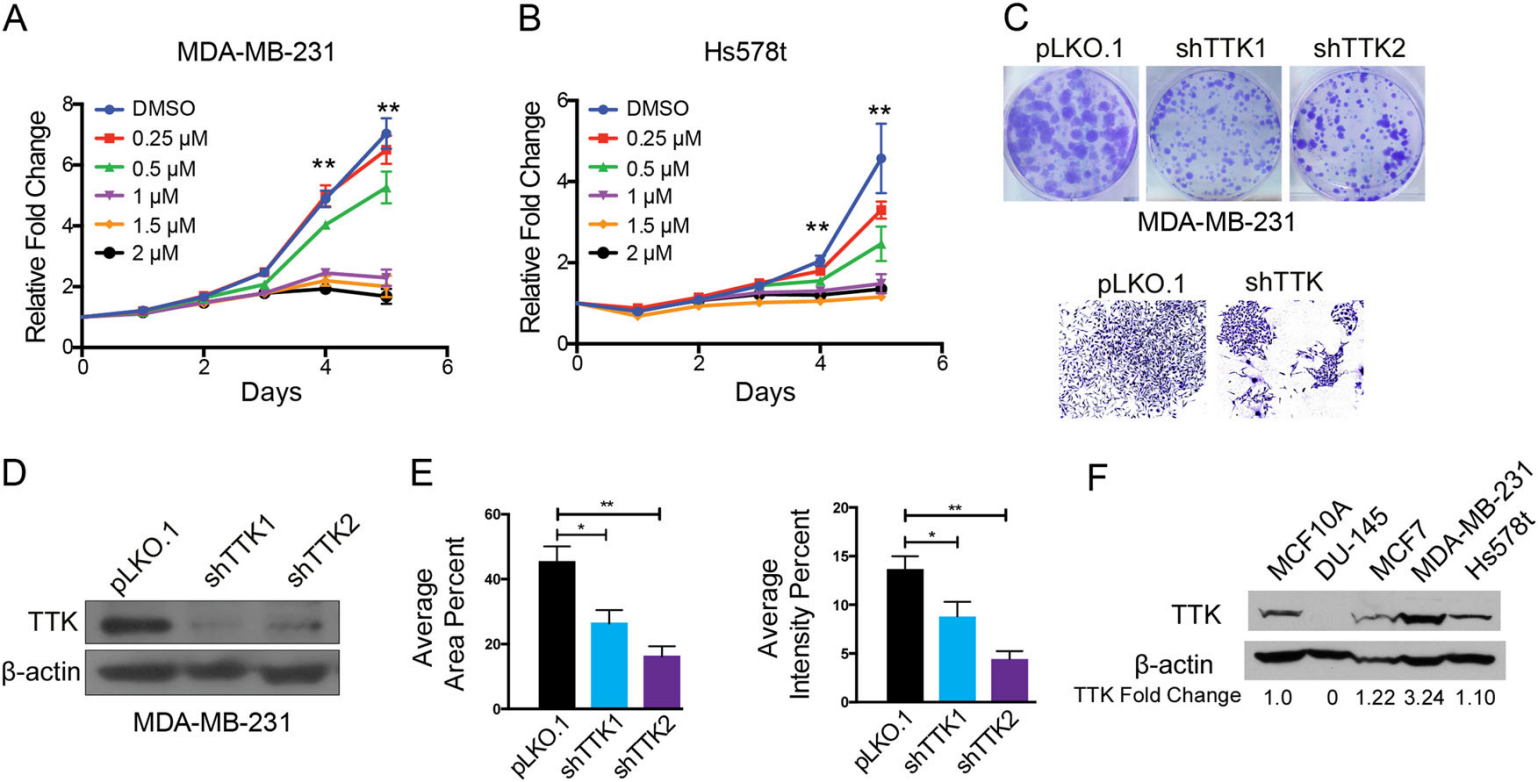
**TTK inhibition suppresses the proliferation of TNBC cells**. **a**, **b** Cell viability in MDA-MB-231 and Hs578t TNBC cells as determined by the SRB assay. **p < .01, **c**–**e** Representative colony formations and validation of stable TTK downregulation via viral delivery of shRNA in MDA-MB-231 cells. *p < .05, **p < .01. **f** Detection of TTK in a panel of cell lines

Previous studies demonstrated TNBC cell proliferation is inhibited by silencing TTK^17^. Cell proliferation in other cancer lines is also inhibited by treatment with a targeted TTK inhibitor, NMS-P715^27^, although TNBC cellular response to NMS-P715 has not been tested. We previously reported silencing TTK could decrease CA in HER2+ breast cancer cell lines. To investigate other functional effects of inhibiting TTK with NMS-P715 in breast cancer, we treated cell lines with inhibitor and measured proliferation using the SRB assay. The most profound changes in cell proliferation caused by NMS-P715 treatment occurred in MDA-MB-231 and Hs578t cell lines (Fig. 2a, b). This finding was interesting, because both of these cell lines belong to the mesenchymal stem cell-like subgroup of TNBC cell lines, and have previously been reported to express higher levels of TTK^20^. To further examine the effects of inhibiting TTK in TNBC, we generated MDA-MB-231 cells stably expressing shRNAs against TTK (shTTK). Significant decrease in colony formation and changes in cell morphology, including fewer cells with protrusions and more-rounded cells were detected over an 11-day period in shTTK cells (Fig. 2c–e), suggesting an epithelial phenotype. We also observed significantly decreased colony formation in Hs578t cells upon transient knockdown of TTK (supplemental Fig. 1). MCF10A mammary epithelial and DU-145 prostate cancer cell lines exhibit lower TTK, while higher TTK was detected in other breast cancer cell lines (Fig. 2f).

### TTK inhibition attenuates the mesenchymal phenotype of MDA-MB-231 and Hs578t TNBC cells

**Fig. 3:**
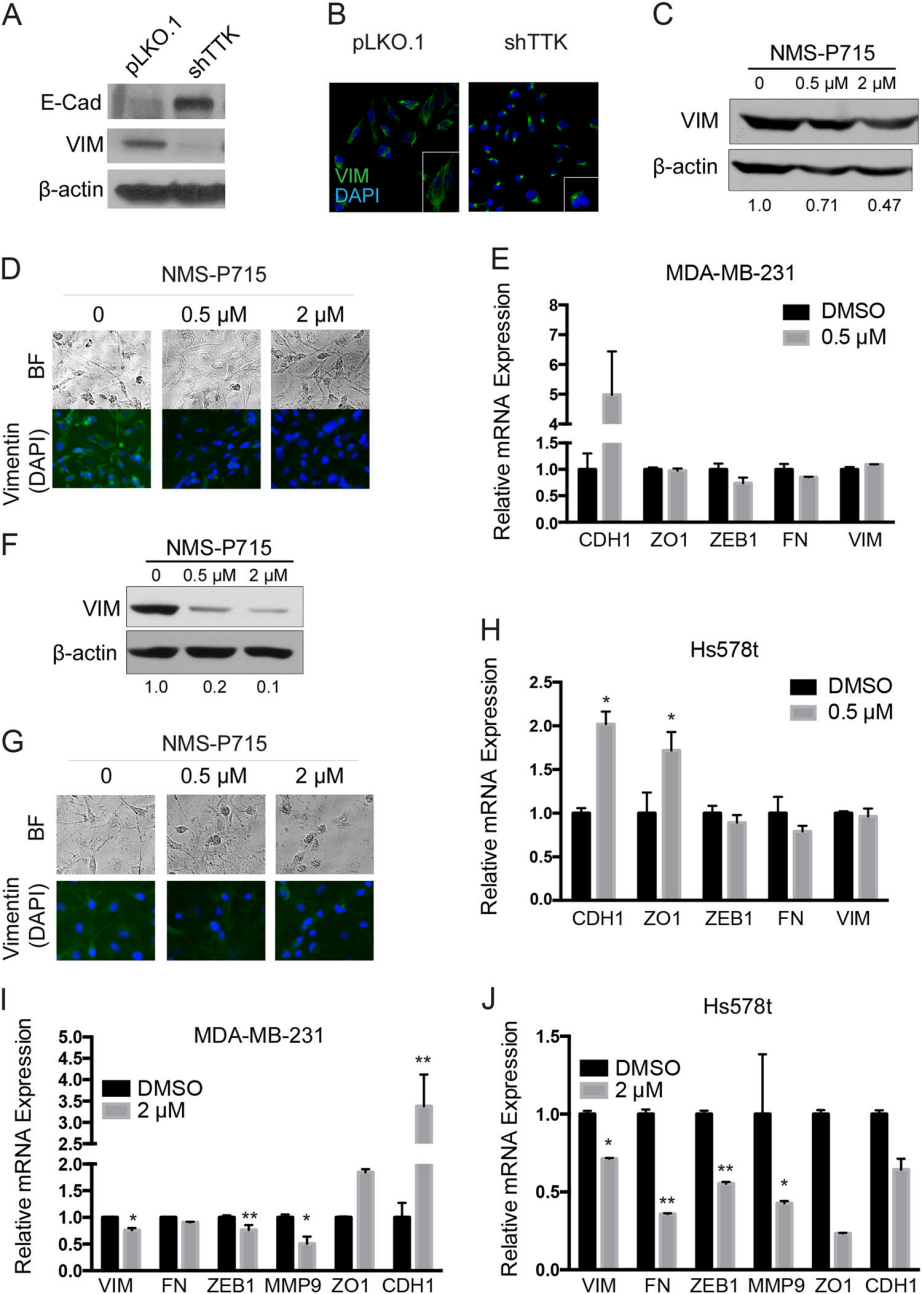
**TTK inhibition attenuates the mesenchymal status of MDA-MB-231 and Hs578t TNBC cells**. **a** Vimentin and E-cadherin protein expression in MDA-MB-231 cells stably expressing the pLKO.1 vector or the shTTK shRNA, as detected by western blotting. **b** Detection of vimentin expression by immunofluorescence staining in MDA-MB-231 cells stably expressing pLKO.1 or shTTK. **c**–**h** Detection of EMT markers in MDA-MB-231 (**c**–**e**, **i**) and Hs578t (**f**–**h**, **j**) TNBC cell lines treated with the NMS-P715 TTK inhibitor by western blotting for protein (**c** and **f**, upper), immunofluorescence staining for vimentin (**d**, **g** lower), and by real-time PCR for mRNA (**e**, **h**–**j**). *p < .05,**p < .01. Bright field (BF) cell morphology is also shown (**d** and **g** upper)

After observing changes in mesenchymal morphology in shTTK cells, we examined the effect of TTK on the epithelial-to-mesenchymal transition (EMT). In MDA-MB-231 cells expressing pLKO.1 control or shTTK, western blotting demonstrated an increased expression of E-cadherin (epithelial marker) and decreased expression vimentin (mesenchymal marker) in the shTTK group (Fig. 3a, b). In transiently transfected Hs578t cells, we observed decreased vimentin, but did not detect changes in E-Cadherin protein (supplemental Fig. 1). Treatment with the NMS-P715 TTK inhibitor also decreased vimentin expression and mesenchymal morphology in both MDA-MB-231 and Hs578t cell lines (Fig. 3c, f). Changes in EMT markers were also measured by rt-PCR. CDH1 mRNA was significantly increased in MDA-MB-231 and Hs578t cells treated with 0.5 μM of NMS-P715 for 24 h, while the mesenchymal markers were not significantly changed in either cell line at 24 or 48 h (Fig. 3e, h). There were moderate decreases in vimentin, fibronectin, ZEB1, and MMP-9 and increases in CDH1 and ZO1 in MDA-MB-231 cells treated with 2 μM NMS-P715 for 48 h (Fig. 3i). Changes in mesenchymal markers were consistent in Hs578t cells, while the changes in epithelial markers differed in this cell line at the 2 μM dose for 48 h (Fig. 3j), which could be attributed to cell density at the time of RNA collection or that these cells express lower TTK than MDA-MB-231. These results suggest that higher TTK expression facilitates mesenchymal signaling in TNBC cells, since these markers were consistently changed in MDA-MB-231 and Hs578t cells at higher doses of NMS-P715.

**Lower KLF5 expression is correlated with TN status of breast cancer and can be increased by TTK inhibition**

**Fig. 4:**
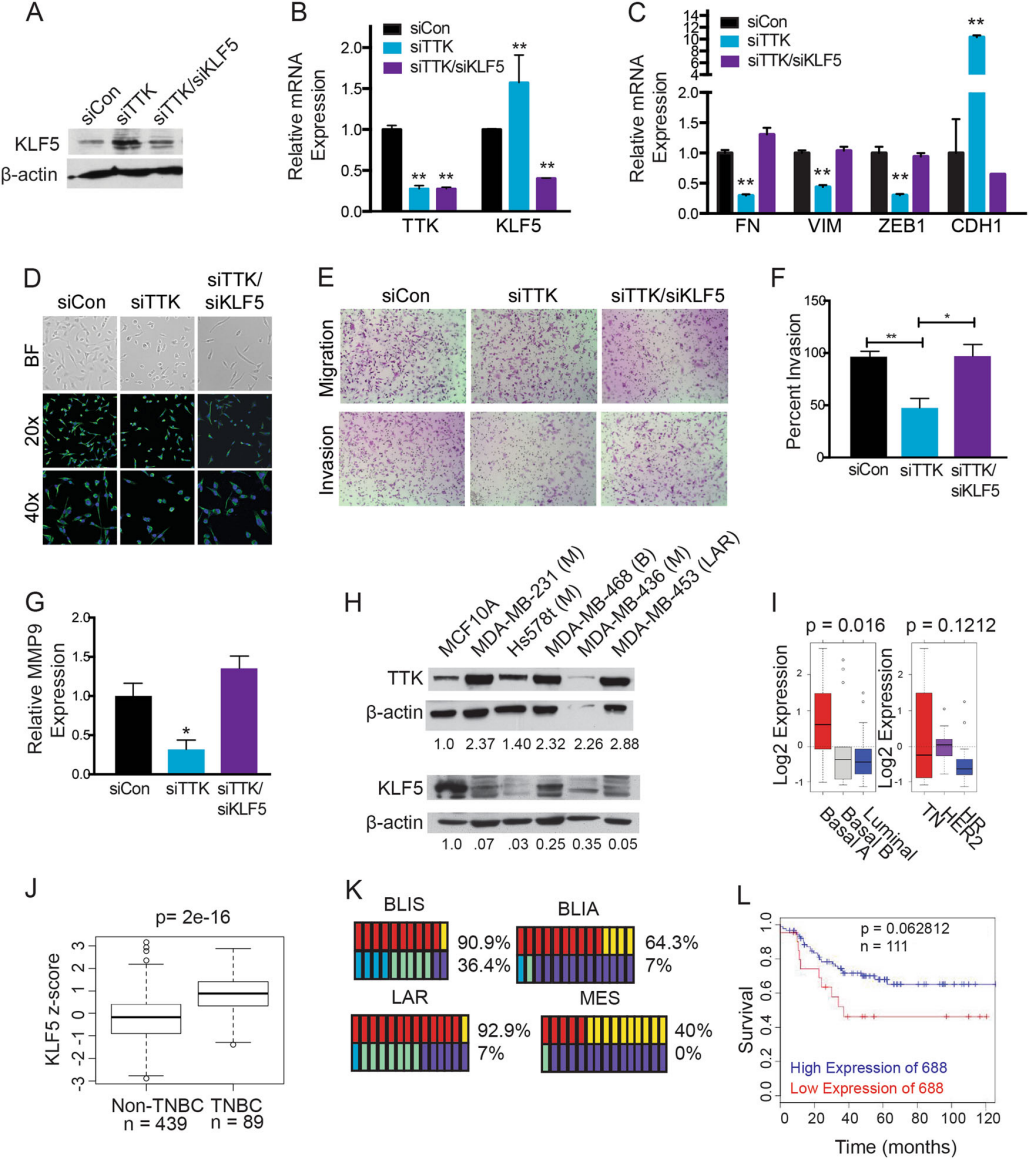
**Downregulation of KLF5 plays a role in TTK-induced EMT and worse patient survival in TNBC**. **a**, **b** Validation of RNAi-mediated TTK and KLF5 knockdown in MDA-MB-231 TNBC cells by western blotting (**a**) and real-time PCR (**b**). **p < 0.01. **c** Expression of EMT markers, as detected by real-time PCR, in MDA-MB-231 cells with the knockdown of TTK or TTK/KLF5. **d** Cellular morphology and detection of vimentin by immunofluorescence staining in MDA-MB-231 cells after TTK or TTK/KLF5 silencing. **e** Representative images of invaded cells in the invasion assay. **f** Quantification of cell invasion. *p < .05 **p < .01. **g** Expression of MMP-9 mRNA as measured by real-time PCR in cells with TTK or TTK/KLF5 knockdown. *p < 0.05 **h** Detection of TTK and KLF5 protein expression by western blotting in different breast cancer cell lines. Subtypes are indicated by M for mesenchymal, B for basal or LAR for luminal androgen receptor. **i** Box plots of KLF5 expression in different subtypes of breast cancer cell lines from the GOBO database. **j** Box plots comparing KLF5 z-scores between non-TNBC subtypes and TNBC patients using data from the TCGA database. **k** Distribution of genes significantly correlated with KLF5 for each TNBC subtype. Top row indicates genes with significant *p* values (<0.05) in red or non-significant *p* values (yellow) for each subtype. Bottom row indicates moderate (blue), weak (green) or very weak (purple) Spearman’s coefficient for each gene. **l** Kaplan–Meier survival analysis for the correlation between KLF5 expression and overall survival in TNBC patients using data from the BreastMark database

Our previous study indicates the KLF5 transcription factor maintains the epithelial phenotype of cells^28^. Mesenchymal TNBC cell lines, including MDA-MB-231 and Hs578T, express a lower level of KLF5 than those of an epithelial phenotype^29^. We therefore tested whether KLF5 plays a role in TTK-promoted EMT in TNBC cells. In MDA-MB-231 cells, silencing TTK increased KLF5 expression at both the RNA and protein levels (Fig. 4a, b), which could be counteracted by silencing KLF5 (Fig. 4a, b). EMT markers were analyzed by rt-PCR, and silencing KLF5 eliminated the effect of silencing TTK on the expression of EMT markers fibronectin (FN), vimentin (VIM), ZEB1, and E-cadherin (CDH1) in MDA-MB-231 cells (Fig. 4c). Morphologically, siTTK cells showed less filamentous vimentin, but silencing KLF5 eliminated the effect of siTTK (Fig. 4d). We also analyzed the functional effects of silencing TTK and KLF5 on the migration and invasion of MDA-MB-231 cells via Boyden chamber assay. Silencing TTK alone did not significantly affect cell migration, but significantly decreased cell invasion through matrigel-coated membranes (Fig. 4e, f); and expression of MMP-9, involved in degradation of the extracellular matrix during cell invasion, was markedly decreased in the siTTK group (Fig. 4g). Similar to its effects on EMT markers, silencing KLF5 eliminated changes in cell invasion and MMP-9 expression caused by TTK silencing (Fig. 4f, g). We determined that silencing KLF5 alone had similar effects as dual silencing of TTK and KLF5 on cell invasion (supplemental Fig. 2). These results indicate that downregulation of KLF5 is required for TTK to induce a mesenchymal phenotype in TNBC cells.

To evaluate the correlations between TTK and KLF5 expression in TNBC, we analyzed TTK and KLF5 protein expression in a panel of cell lines. In MCF10A cells, TTK levels were low, while KLF5 was expressed at moderate levels. Meanwhile, the TNBC cell lines used expressed higher levels of TTK but lower KLF5 compared to MCF10A cells (Fig. 4h). We analyzed a small subset of additional TNBC cell lines, and observed moderate KLF5 expression in the TNBC cells line MDA-MB-468 that do not belong to the MSL subgroup. We also analyzed a small cohort of primary breast tumor tissues for TTK and KLF5 expression. While we did not detect significant changes in TTK expression in this cohort between subtypes, we observed changes in KLF5 localization between subtypes. For the TNBC samples in this cohort with TTK expression we observed low KLF5 levels, and where KLF5 was present, it was not localized in the nucleus like in the normal samples (Supplemental Fig. 4).

In the GOBO database, lower KLF5 expression was significantly correlated with the Basal B subtype (which includes MDA-MB-231 and Hs578t) and the overall TN status but not with the Basal A status in breast cancer cell lines (Fig. 4i). In the TCGA database, higher KLF5 expression correlated with the TN status in breast cancer (Fig. 4j). TNBCs in the TCGA database have not been classified into subgroups of Basal A, Basal B and Mesenchymal stem cell-like, which prevented us from testing which subtype is correlated with the highest KLF5 expression. To address this, we selected genes overexpressed in different subtypes of TNBC identified in a previous study^30^, and analyzed if their expression correlates with KLF5 expression in the TCGA dataset. In this analysis, the basal-like-immunosuppressed group and luminal androgen receptor subtypes had the highest percentages of genes correlated with KLF5 expression (90.9 and 92.9%, respectively), with the basal-like immunosuppressed group having the highest number of genes with Spearman’s coefficients over 0.40 (36.4%), which is indicative of a moderate correlation (Fig. 4k). The basal-like immunoactivated group had 64.3% of genes associated with KLF5 expression, but only 7% had moderate Spearman’s coefficients. The mesenchymal subgroup had only 40% of genes associated with KLF5 expression, none of which had strong Spearman’s coefficients. These results further suggest that there is minimal correlation between high KLF5 expression within mesenchymal TNBCs as compared to other subtypes of TNBC.

In the BreastMark cohort, lower KLF5 expression significantly correlated with decreased overall survival in TNBC patients (Fig. 4l). Together, these results suggest that high TTK levels could be correlated with decreased KLF5 in mesenchymal TNBC, partially due to changes in localization of the transcription factor.

### TTK inhibition attenuates TGF-β signaling and miR-21 expression

**Fig. 5:**
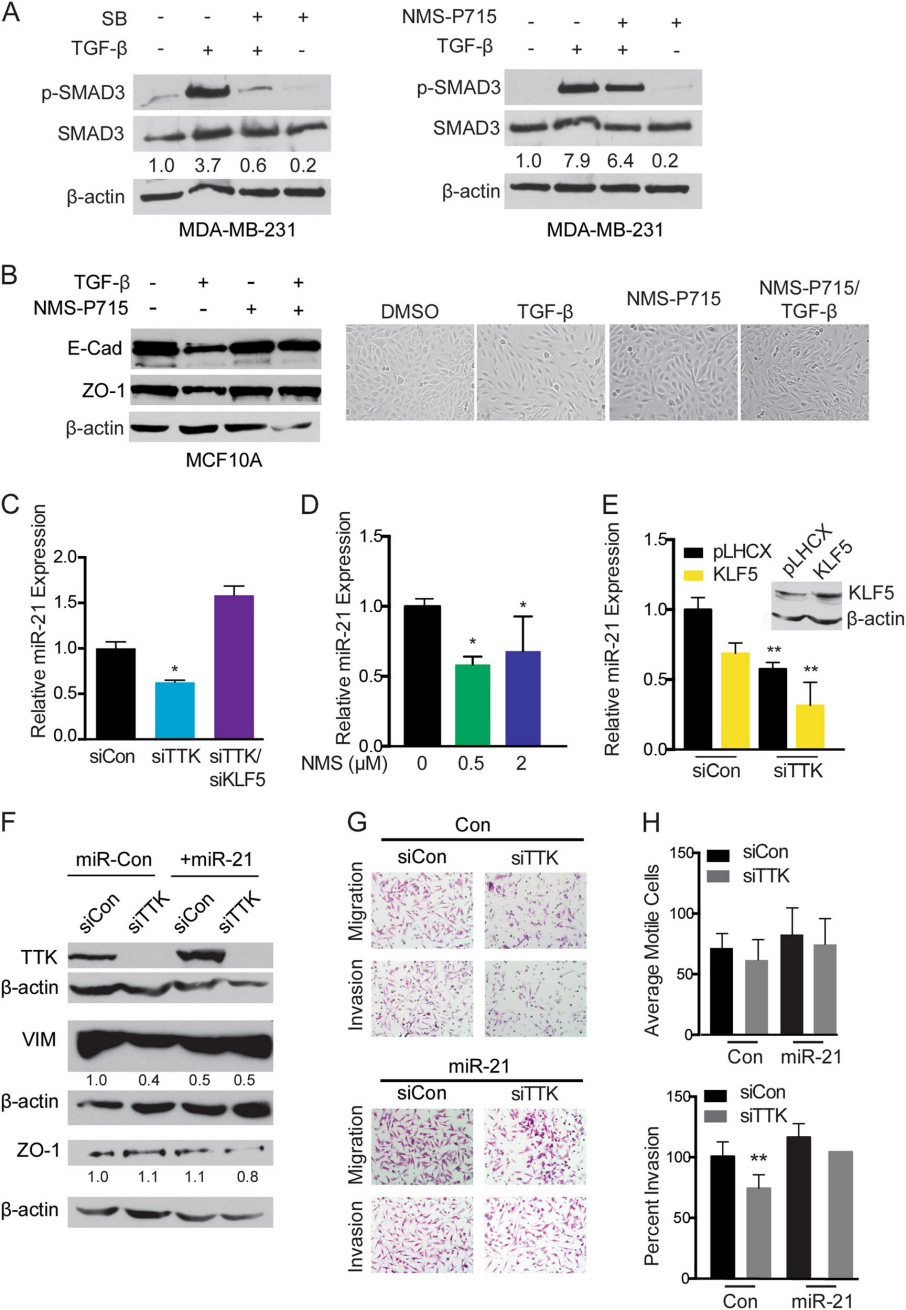
**Effects of TTK inhibition on TGF-β signaling and miR-21 expression in MDA-MB-231 breast cancer cells**. **a** Comparison of the SB505124 TGF-β inhibitor and the NMS-P715 TTK inhibitor for their effects on Smad3 phosphorylation in MDA-MB-231 cells, as measured by western blotting. **b** Detection of EMT markers E-cadherin and ZO1 in and images of MCF10A mammary epithelial cells following treatments with TGF-B, the NMS-P715 TTK inhibitor, and their combination. **c**–**e** Detection of mir-21 expression by real-time PCR in MDA-MB-231 cells with the knockdown of TTK or TTK/KLF5 by siRNA (**c**), treatment with the NMS-P715 TTK inhibitor (**d**), and lentivirus mediated KLF5 overexpression (**e**). *p < .05, **p < .01. **f** Protein expression of EMT markers in MDA-MB-231 cells with siRNA knockdown of TTK and miR-21 overexpression. **g**, **h** Representative images of invaded cells (**g**) and their quantification in motility and invasion assays. **p < .01

TGF-β signaling is the most potent of the signaling pathways known to induce EMT, and KLF5 was previously demonstrated to participate in TGF-β signaling^31,32^. We tested if TGF-β signaling is involved in the inverse relationship between TTK and KLF5 and their role in EMT regulation in TNBC cells. TTK has been reported to regulate the TGF-β signaling pathway, as TTK modulates SMAD3 phosphorylation^33–35^. In MCF10A cells, which clearly respond to TGF-β, we confirmed that TGF-β induced EMT, as indicated by morphological and EMT marker changes (Fig. 5b). This induction of EMT was mildly prevented when MCF10A cells were treated with NMS-P715 in conjunction with TGF-β (Fig. 5b). We further tested the effect of TTK inhibition on SMAD3 phosphorylation in MDA-MB-231 cells, and found inhibition of TTK by NMS-P715 slightly decreased TGF-β-induced Smad3 phosphorylation (Fig. 5a).

Micro-RNAs are important mediators of EMT, and micro-RNA 21 (miR-21), a downstream target of TGF-β involved in EMT, is regulated by TTK in glioblastoma cells^34^. To test if miR-21 is regulated by TTK in TNBC cells, miR-21 expression was measured in MDA-MB-231 cells after silencing TTK or NMS-P715 treatment. miR-21 expression was decreased by siTTK and simultaneous knockdown of KLF5 rescued the downregulation of miR-21 by siTTK (similar to siKLF5 alone, supplemental Fig. 3, Fig. 5c). miR-21 expression was also decreased in NMS-P715 treated MDA-MB-231 cells (Fig. 5d). Ectopic expression of KLF5 decreased miR-21 expression in MDA-MB-231 cells, which was augmented by TTK silencing (Fig. 5e). These results suggest TTK modulates miR-21 expression in TNBC cells, and the effect of TTK is likely dependent on KLF5 downregulation. Functionally, ectopic expression of miR-21 partially attenuated the effects of TTK silencing on EMT and invasion in MDA-MB-231 cells (Fig. 5g, h), further suggesting a partial role of miR-21 in TTK-mediated EMT in TNBC cells (Fig. 5f).

### Inhibitory effects of TTK on KLF5-activated miR-200 expression

The miR-200 family of micro-RNAs regulates EMT in a feedback loop with ZEB1 and ZEB2, which bind promoter regions of miR-200s to prevent their transcription and repress the epithelial phenotype^36^. In a previous study, we showed that KLF5 maintains the epithelial phenotype by directly binding to the GC boxes in the miR-200 gene promoters to activate their expression^28^. In the same study, we also showed that TGF-β–induced EMT requires KLF5 downregulation and the subsequent downregulation of miR-200s^28^. Finally, miR-200 family members were upregulated in MDA-MB-231 cells following ectopic KLF5 expression^28^. We therefore tested whether the inhibitory effect of TTK on KLF5 expression also leads to the repression of KLF5-activated miR-200s during TTK-induced EMT in TNBC cells.

**Fig. 6:**
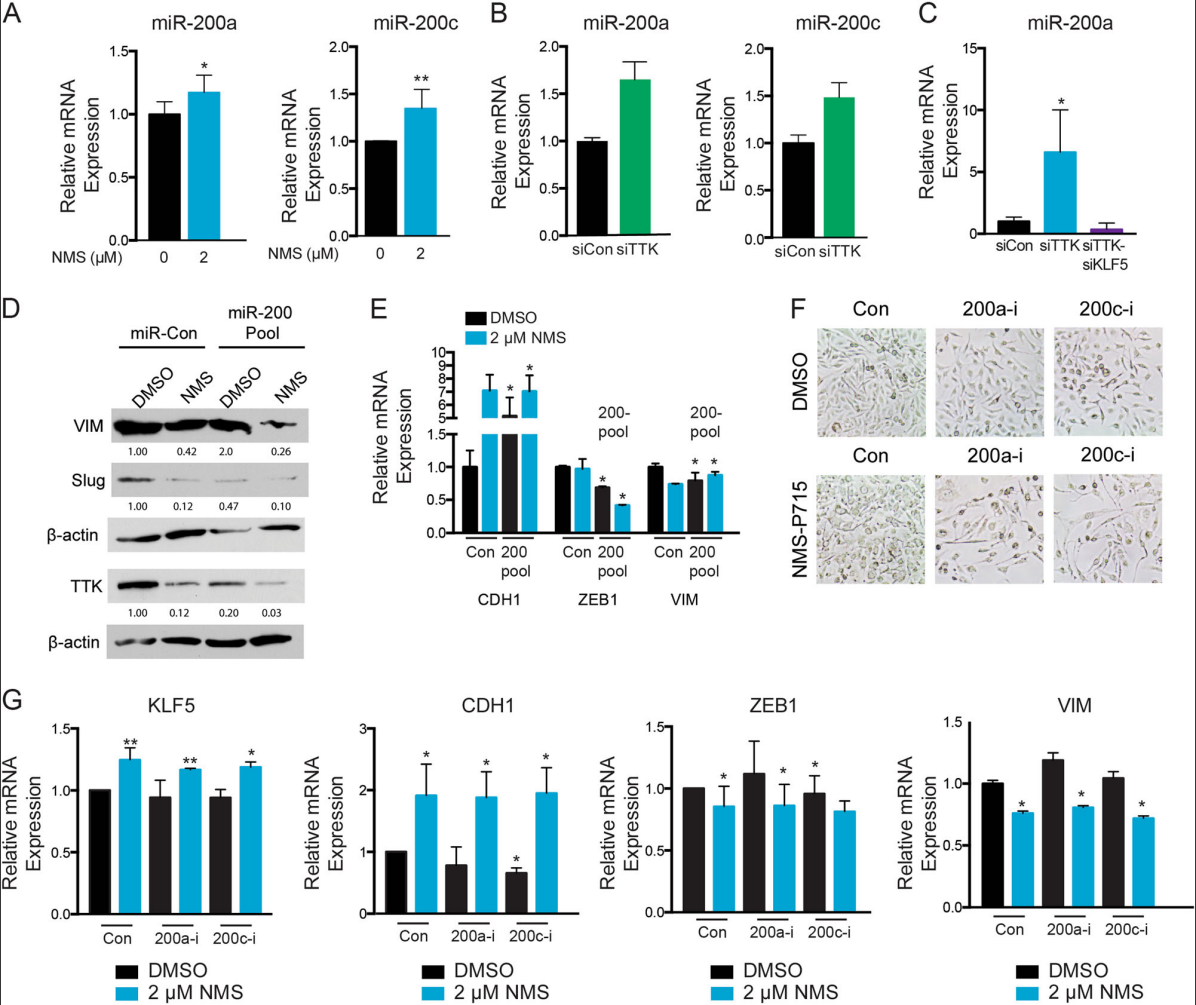
**TTK inhibition upregulates the expression of miR-200a and miR-200c to attenuate EMT in MDA-MB-231 cells**. **a**, **b** Detection of miR-200a and miR-200c expression by real-time PCR in MDA-MB-231 cells treated with the NMS-P715 TTK inhibitor (**a**) or TTK and KLF5 siRNA (**b**, **c**). **d**, **e** Expression of EMT markers in MDA-MB-231 cells treated with NMS-P715 in combination with pools of miR-200 mimics. **f**, **g** Representative images of cells (**f**) and detection of mRNA expression of KLF5, CDH1, ZEB1, and VIM by real-time PCR (**g**) in MDA-MB-231 cells treated with NMS-P715 in the presence or absence of inhibitors against miR-200a or miR-200c. **p < .01; *p < .05

In MDA-MB-231 cells, we observed significantly increased miR-200a and miR-200c expression following after NMS-P175 treatment (Fig. 6a). Silencing TTK showed similar effects on the expression of miR-200a and miR-200c in MDA-MB-231 cells (Fig. 6b). Expression of miR-200a and miR-200c was also decreased by silencing both TTK and KLF5 and similar results were observed in the siKLF5 alone group (Supplemental Fig. 3, Fig. 6c).

Functionally, we tested if combining TTK inhibition with miR-200 overexpression attenuates the effect of TTK on EMT in TNBC cells. Cells were transfected with a pool of miRNA mimics containing miR-200a, miR-200b, and miR-200c and treated with the NMS-P715 inhibitor. NMS-P715 treatment alone downregulated mesenchymal markers vimentin, ZEB1 and Slug, while upregulating epithelial marker CDH1 (Fig. 6d, e). Overexpression of miR-200 family members alone had similar effects as NMS-P715 treatment alone. Interestingly, combination of miR-200 overexpression with NMS-P715 treatment further downregulated vimentin, Slug, ZEB1 and upregulated CDH1 compared to the other groups (Fig. 6d, e).

We also transfected cells with inhibitors against miR-200a and miR-200c to determine if inhibiting these microRNAs attenuates the effect of TTK inhibition on EMT changes. Morphological changes were observed in MDA-MB-231 cells treated with inhibitors of miR-200a and miR-200c, indicative of a mesenchymal phenotype, which were not prevented when these groups were treated with NMS-P715 (Fig. 6f). Downregulation of vimentin by TTK inhibition in MDA-MB-231 cells was not reversed by miR inhibition (Fig. 6g). Inhibiting the miRs also did not reverse the upregulation of KLF5 and CDH1 and downregulation of ZEB1 induced by TTK inhibition (Fig. 6e), indicating that silencing miR-200a or miR-200c does not completely reverse the effect of TTK inhibition on EMT.

## Discussion

### TTK overexpression leads to the mesenchymal phenotype of triple negative breast cancer

Previous studies characterized the role of TTK in mitotic regulation^37–40^, and overexpression of TTK facilitates genomic instability in cancer. In addition to promoting genomic instability and aneuploidy, TTK promotes cancer cell proliferation and invasion^22^. On the basis of these tumor-promoting roles, TTK is an attractive therapeutic target. However, the cellular mechanisms by which TTK facilitates these processes are unclear. Some proposed pathways for TTK promoting cell survival and invasion include AKT signaling and the regulation of miR-21 via TGF-β. In this study, we examined the correlations between TTK expression and clinical characteristics of breast cancer using publically-available databases, and confirmed the correlation between high levels of TTK expression with adverse features (Fig. 1). Further supporting an oncogenic function of TTK, inhibition of TTK activity by either RNAi or NMS-P715 significantly suppressed cell proliferation or survival (Fig. 2).

EMT is associated with aggressive cancer behaviors including invasion and metastasis. We report for the first time that TTK promotes the mesenchymal status of TNBC cells, because silencing TTK or inhibiting its activity caused a reversion from the mesenchymal to epithelial phenotype, including decreased mesenchymal markers and invasion, and increased expression of epithelial markers (Figs. 3 and 4). Maintaining a mesenchymal morphology further supports a role of TTK in the progression of TNBC.

### Maintenance of EMT by TTK involves the downregulation of KLF5 and its transcriptional targets miR-200s

KLF5 maintains the epithelial phenotype by activating miR-200s and downregulation of KLF5 is essential for TGF-β to induce EMT^28^. KLF5 promotes proliferation in some TNBC cells^41,42^. KLF5 function could be context-dependent, including a role in TTK-mediated EMT due to specific cell signaling, tumor microenvironment, and the subtype of TNBCs. In this regard, higher KLF5 expression associated with the TN status of breast cancer, and more specifically, KLF5 expression was higher in the basal A than basal B subtype of TNBC (Fig. 4h), which inversely correlated with TTK expression (Fig. 1d). In MDA-MB-231 mesenchymal TNBC cells, KLF5 expression was lower, which was partially attributed to increased TTK expression, since inhibiting TTK by RNAi upregulated KLF5 expression (Fig. 4a). How TTK downregulates KLF5 remains unknown, but could be related to TGF-β signaling, as TGF-β downregulates KLF5^28^ and TTK inhibition reduced the activation of SMAD3 by TGF-β (Fig. 5a), potentially impacting the assembly of the transcriptional complex for KLF5 or altering the cellular localization and function of KLF5.

**Fig. 7:**
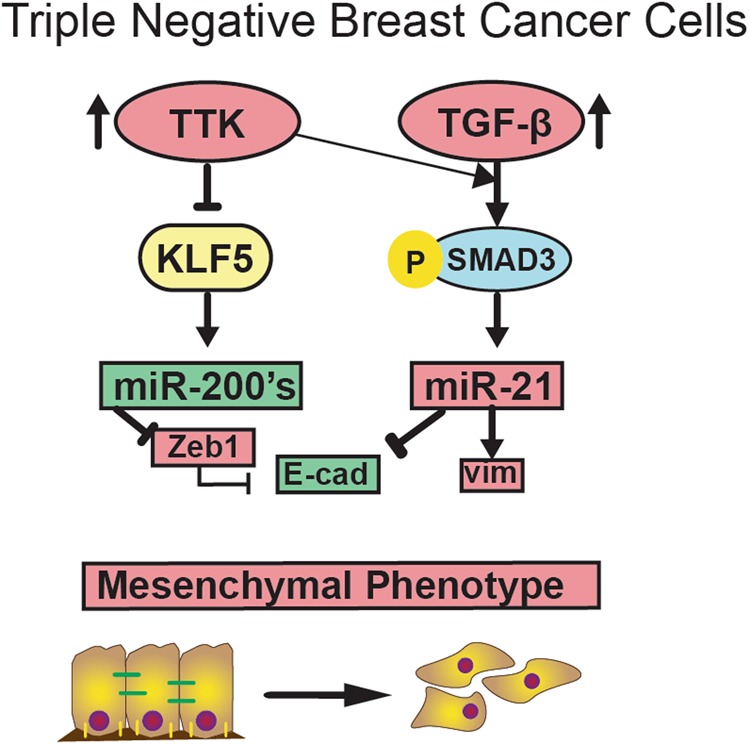
**A potential model for how TTK promotes EMT in TNBC cells**. In normal breast cells, the TTK expression level is low while that for KLF5 is higher, and, KLF5 induces the transcription of miR-200 family members and represses miR-21 expression, leading to the epithelial phenotype. However, in TNBC cells, higher TTK expression leads to decreased KLF5 expression, resulting in the downregulation of miR-200s and upregulation of miR-21 and the induction of EMT in TNBC cells

KLF5 downregulation by TTK contributes to TTK-mediated EMT, as knockdown of KLF5 by RNAi attenuated the effect of TTK inhibition of EMT (Fig. 4a–e). Additionally, inhibiting TTK upregulates the expression of miR-200a and miR-200c, which are well-established repressors of EMT that downregulate ZEB1 and ZEB2 expression, and activated by KLF5 to suppress EMT and maintain the epithelial phenotype^28^ (Fig. 6). In parallel, miR-200 mimics appeared to also downregulate TTK expression. Potential mechanisms could involve changes in TTK protein stability or mitotic activity induced by the miR-200′s presence in the cell. In addition to supporting a role of TTK in EMT, these findings also provide a mechanism for TTK-induced EMT, whereby higher TTK expression suppresses KLF5 expression in mesenchymal TNBC cells, and decreased KLF5 expression reduces miR-200 expression and subsequent upregulation of mesenchymal genes and downregulation of epithelial genes (Fig. 7).

### TTK likely interacts with the TGF-β signaling pathway to induce EMT

Smad3 is a key downstream effector of TGF-β, the most potent known inducer of EMT^43^. TGF-β signaling has also been demonstrated to be more active and associated with worse patient survival in TNBC^44^. TTK preferentially phosphorylates Smad3 but not Smad2 in the SSXS motif, which is similar to TGF-β^33^. We found that TTK modulates TGF-β signaling, as TTK inhibition decreased SMAD3 phosphorylation induced by TGF-β (Fig. 5a).

In addition to miR-200a and miR-200c, TTK indirectly regulates the expression of miR-132^45^ and miR-21^34^. MiR-21 is a downstream target of TGF-β that is transactivated to promote the mesenchymal cell status and considered an onco-miR^46–48^. TTK upregulates miR-21^34^, and we demonstrated in TNBC cells, TTK also upregulates miR-21 expression, as inhibition of TTK by siRNA or inhibitor decreased miR-21 expression, and restoration of miR-21 moderately prevented the effect of TTK on EMT marker expression (Fig. 5). Therefore, promotion of TGF-β-induced miR-21 expression is likely another mechanism by which TTK promotes EMT.

Analysis using the TargetScan online resource, which predicts biological targets of miRNAs^49^, demonstrated KLF5 also has a target sequence for miR-21. Together with the observation that TTK inhibition decreased miR-21 expression while increasing KLF5 expression, TTK-upregulated miR-21 is likely a mechanism underlying the TTK-mediated downregulation of KLF5 and induction of EMT (Fig. 7).

In addition to downregulating KLF5 and miR-200 expression during EMT induction^28^, TGF-β induces KLF5 acetylation, and acetylated KLF5 binds with Smads to mediate TGF-β’s inhibitory function in cell proliferation and tumor growth^32,50^. It remains unknown whether acetylated KLF5 plays a role in TTK-induced EMT.

### Clinical implications of TTK-induced EMT in TNBC

This study highlights the role of TTK in EMT induction and mesenchymal TNBC involving TGF-β signaling, KLF5, and miRNAs. Targeting TTK with small-molecule inhibitors has become a viable therapeutic approach in recent years, since aneuploid tumors are usually more sensitive to TTK inhibition^51^. Targeting TTK could also improve existing therapeutic strategies such as taxane therapy or radiation treatment^24,52–54^. For example, targeting TTK appears to radiosensitize glioblastoma^34^, and enhance the efficacy of docetaxel in the treatment of breast cancer^24^.

However, signaling pathways associated with high TTK expression have not been comprehensively characterized, and whether and how TTK contributes to various disease statuses remains poorly understood. This study provides evidence for the role of TTK in EMT and subsequent tumor progression and metastasis. It is thus likely that targeting TTK could attenuate tumor progression.

The current study also provides novel information about the signaling pathways associated with TTK overexpression and tumor aggressiveness in TNBC, including its interaction with TGF-β signaling and its regulation of the EMT regulators KLF5, miR-21, and miR-200s. Such mechanistic insight into how TTK mediates EMT could facilitate the study of TTK expression as a biomarker for tumor aggressiveness in the context of TGF-β signaling and treatment responses in breast cancer patients.

## Methods

### Bioinformatic data and analyses

GOBO and BreastMark online databases were used to analyze the expression levels of TTK and KLF5 in different molecular and clinical subtypes of breast cancer. In the BreastMark database, we analyzed overall survival as the clinical endpoint. RNAseq data was obtained from TCGA to analyze TTK and KLF5 z-scores in breast cancer patients. Patients were categorized by subtype and z-scores were analyzed using the RStudio software (http://www.rstudio.com/).

### Cell culture, inhibitor treatment, and transfection

MCF10A, MDA-MB-231, Hs578t and other cells were obtained from ATCC and cultured as previously described^28,55^. NMS-P715 (TTK inhibitor) was purchased from Millipore and TGF-β treatment as previously described^28^. Scrambled control and custom TTK siRNA duplexes were purchased from IDT (sequences described in^10^. KLF5 custom siRNA’s were previously described^28^. MiR-21 mimics and negative controls were purchased from Thermo. MiR-200 mimics and inhibitors were purchased from RiboBio. Transfections were performed using JetPrime Polyplus, per manufacturer’s instructions.

### Viral infection

HEK293T cells were cultured to 80% confluence and transfected with control shRNA pLKO.1 or pLHCX or TTK shRNA or flag-tagged KLF5 constructs. Lipofectamine 2000 was used per manufacturer’s instructions. Media was collected 48 h post-transfection. Target cells were infected with viruses + 8 μg/mL polybrene at 60% confluence. Stable cells expressing shTTK or KLF5 constructs were selected with 2 μg/mL puromycin or 200 μg/mL hygromycin post-infection.

### Colony formation assays

Colony formation assays were performed as previously described^55^. Colonies (clusters of >50 cells) were counted and colony intensities were calculated via ImageJ.

### SRB viability assay

Cells were plated at a density of 1500 cells per well into 96-well plates in triplicate and cultured overnight before NMS-P715 treatment. Cells were fixed with 10% TCA for 1 h before adding 0.05% SRB dye for 30 min then dissolving in 10 mM Tris-HCL for 30 min. Cell viability was quantified via 510 nm absorbance.

### Western blot analysis

Cells were rinsed with PBS and lysed directly with Laemmli buffer containing β-mercaptoethanol. Total protein lysates were separated by SDS-PAGE, transferred to nitrocellulose membranes and subjected to western blot analysis. Primary antibodies for EMT markers (#9782), TTK (#5469), and secondary rabbit antibody (#7074) were obtained from Cell Signaling. The β-actin antibody was from Sigma (A2006), and the antibody for KLF5 was generated and described in a previous study^56^.

### Immunofluorescence microscopy

Immunofluorescent staining was performed as previously described^28^ and imaged on a Zeiss LSM 510.

### Quantitative RT-PCR

Reverse transcription PCR was performed as previously described^28^ and quantitative real-time PCR was performed using Takara SYBR green on a 7500 Fast qPCR machine from Applied Biosystems. For micro-RNA PCR, RNA was isolated using the miRNeasy mini kit from Qiagen. CDNA was synthesized using the TaqMan MicroRNA Reverse Transcription Kit from ThermoFisher. rt-PCR was conducted with Taqman Universal Master Mix and custom Taqman probes for miR-21, miR-200s, and U6 non-coding RNA. The 2^-ΔΔCt^ method was used to analyze and quantify mRNA and miRNA levels. Values were normalized against actin or U6 as internal controls then compared to experimental controls to obtain differences in fold changes. Primer sequences for PCR were: *ZEB1*: 5′-TGCACTGAGTGTGGAAAAGC-3′ and 5′-TGGTGATGCTGAAAGAGACG-3′; *ZO1*: 5′-CCCCACTCTGAAAATGAGGA-3′ and 5′-ACAGCAATGGAGGAAACAGC-3′; *CDH1*: 5′-TGAAGGTGACAGAGCCTCTGGAT-3′ and 5′-TGGGTGAATTCGGGCTTGTT-3′; *FN1*: 5′-CCATAAAGGGCAACCAAGAG-3′ and 5′-ACCTCGGTGTTGTAAGGTGG-3′; *VIM*: 5′-CAGGCGATATATTACCCAGGCAAG-3′ and 5′-CTTGTAGGAGTGTCGGTTGTTAAG-3′; *MMP9*: 5′-ACGTGAACATCTTCGACGCCATC-3′ and 5′-TCAGAGAATCGCCAGTACTTCCC-3′; *KLF5*: 5′-AAGGAGTAACCCCGATTTGG-3′ and 5′-CAGCCTTCCCAGGTACACTT-3′; *TTK*: 5′-CGCAGCTTTCTGTAGAAATGGA-3′ and 5′-GAGCATCACTTAGCGGAACAC-3′; *GAPDH*: 5′-GGTGGTCTCCTCTGACTTCAACA-3′ and 5′-GTTGCTGTAGCCAAATTCGTTGT-3′.

### Boyden chamber assays

For boyden chamber assays, 35,000 cells/well were plated on top of a transwell chamber (matrigel-coated for invasion assays) and incubated for 24 h. Non-invading cells in the upper chambers were removed, while cells that migrated to the lower chamber were fixed with 0.5% crystal violet prepared in methanol. Cells were counted in 12 fields/group. Average motile cells or percent invaded cells were calculated for three independent experiments.

### Immunohistochemistry Staining

Breast cancer tissue arrays (Cat. #BRC961) were purchased from US Biomax and stained for TTK and KLF5 as previously described^57^. Four images were taken/sample.

### Statistical analysis

Graphpad Prism was used to analyze data. Values are represented as mean + /− SEM for *n* = 3 experiments and considered significant if *p* < 0.05. Student’s *t* test (unpaired, two-tailed) was used to compare differences between groups. For TCGA data acquired from online repositories, the Hmisc package (https://CRAN.R-project.org/package = Hmisc) was used to analyze correlations of gene expression in each population.

## Electronic supplementary material

Supplemental Figure Legends

Supplemental Figure 1

Supplemental Figure 2

Supplemental Figure 3

Supplemental Figure 4
