# Supplementary material for: TTK promotes mesenchymal signaling via multiple mechanisms in triple negative breast cancer
Source: Oncogenesis. 2018 Sep 12;7(9):69. doi: 10.1038/s41389-018-0077-z (PMC6133923; doi:10.1038/s41389-018-0077-z)
Supplement: Supplementary file 1 — Supplemental Figure Legends [file 41389_2018_77_MOESM1_ESM.doc]

**Supplemental Figure 1**: Transient TTK inhibition suppresses proliferation and mesenchymal phenotype of TNBC cells **(a)** Validation of TTK knockdown in Hs578t cells and vimentin protein levels. **(b,c)** Representative colony formations in Hs578t cells and quantifications,**, p<.­­­01**. (d,e)** Cell viability in MDA-MB-231 and Hs578t cells transiently silenced for TTK as measured by SRB assays, *, p<.05.

**Supplemental Figure 2**: Effects of single knockdown of KLF5 in MDA-MB-231 cells. **(a-b)** Representative images of invaded cells in the invasion assay and quantification of cell invasion. *, p<.­­­05. (c) Validation of RNAi-mediated TTK and KLF5 knockdown, and CDH1 expression in MDA-MB-231 TNBC cells by realtime PCR**.**

**Supplemental Figure 3:** Effects of single knockdown of KLF5 on miR-21 and miR-200c expression in MDA-MB-231 cells. **(a,b)** Detection of mir-21 and miR-200c expression by real-time PCR in MDA-MB-231 cells with the knockdown of TTK, KLF5 or TTK/KLF5 by siRNA *, p<.­­­05.

**Supplemental Figure 4:** Immunohistochemistry staining of TTK and KLF5 in breast cancer tissue array.
