# Supplementary figures and images for: TTK promotes mesenchymal signaling via multiple mechanisms in triple negative breast cancer
Source: Oncogenesis. 2018 Sep 12;7(9):69. doi: 10.1038/s41389-018-0077-z (PMC6133923; doi:10.1038/s41389-018-0077-z)

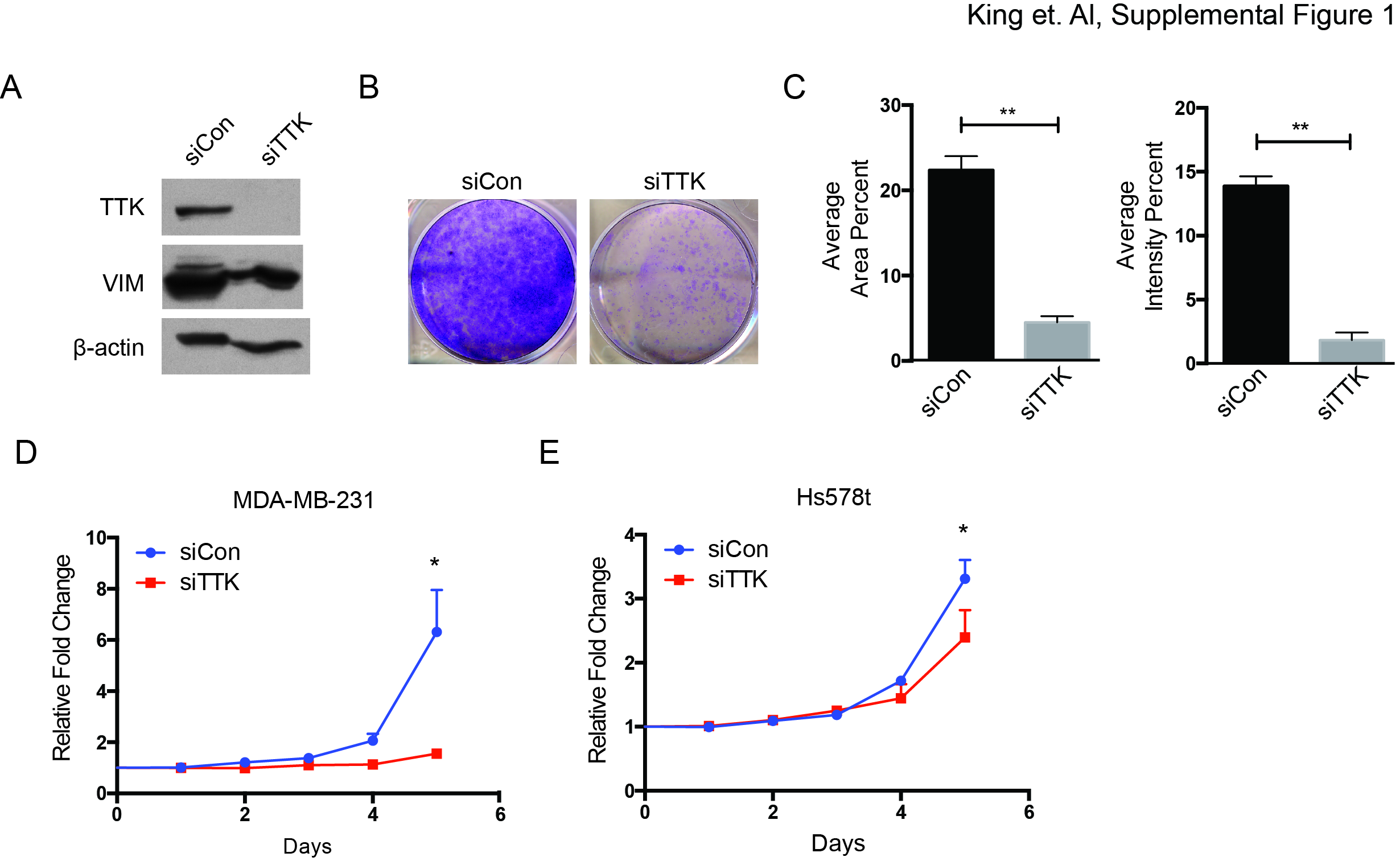

Supplement: Supplementary file 2 — Supplemental Figure 1 [file 41389_2018_77_MOESM2_ESM.tif]

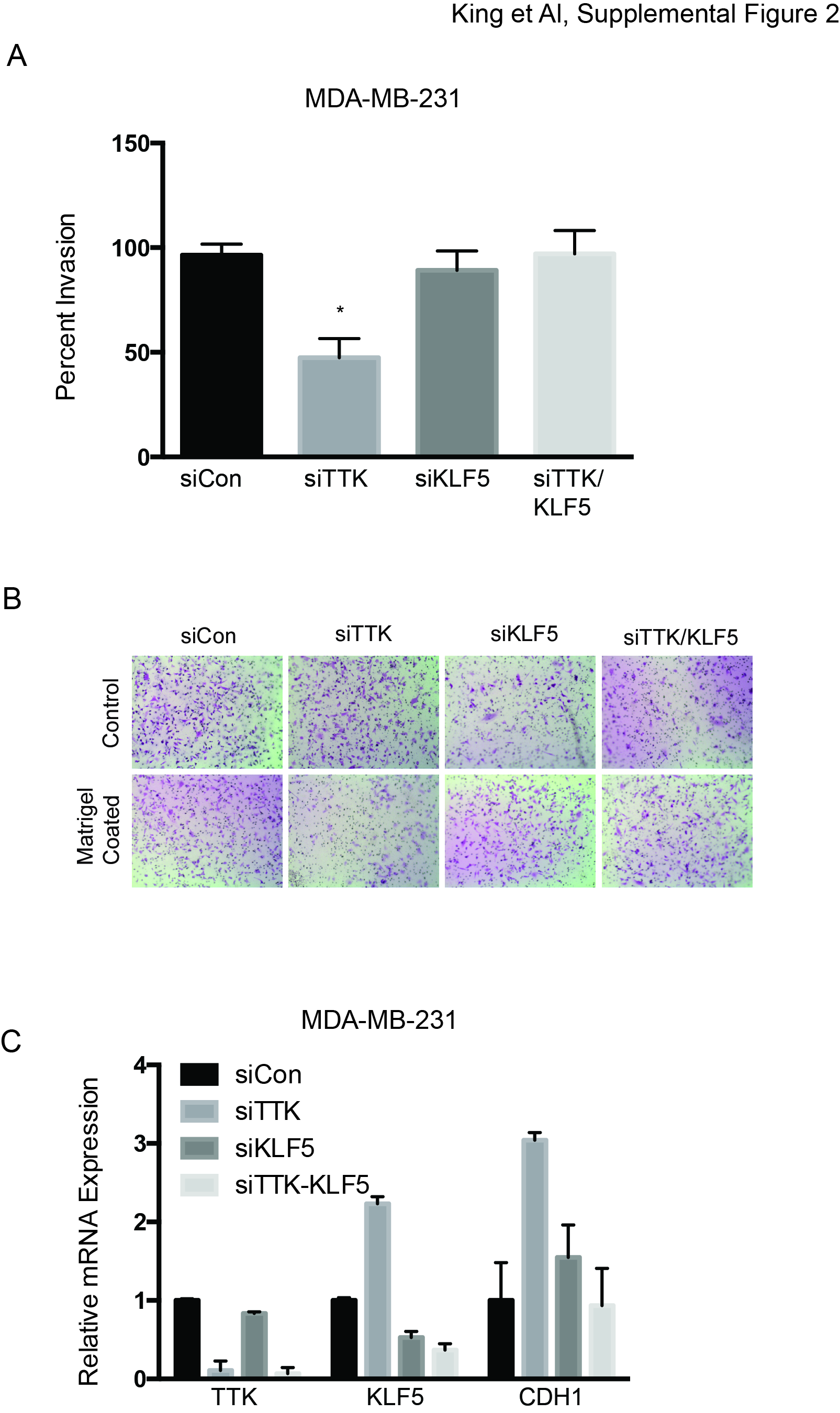

Supplement: Supplementary file 3 — Supplemental Figure 2 [file 41389_2018_77_MOESM3_ESM.tif]

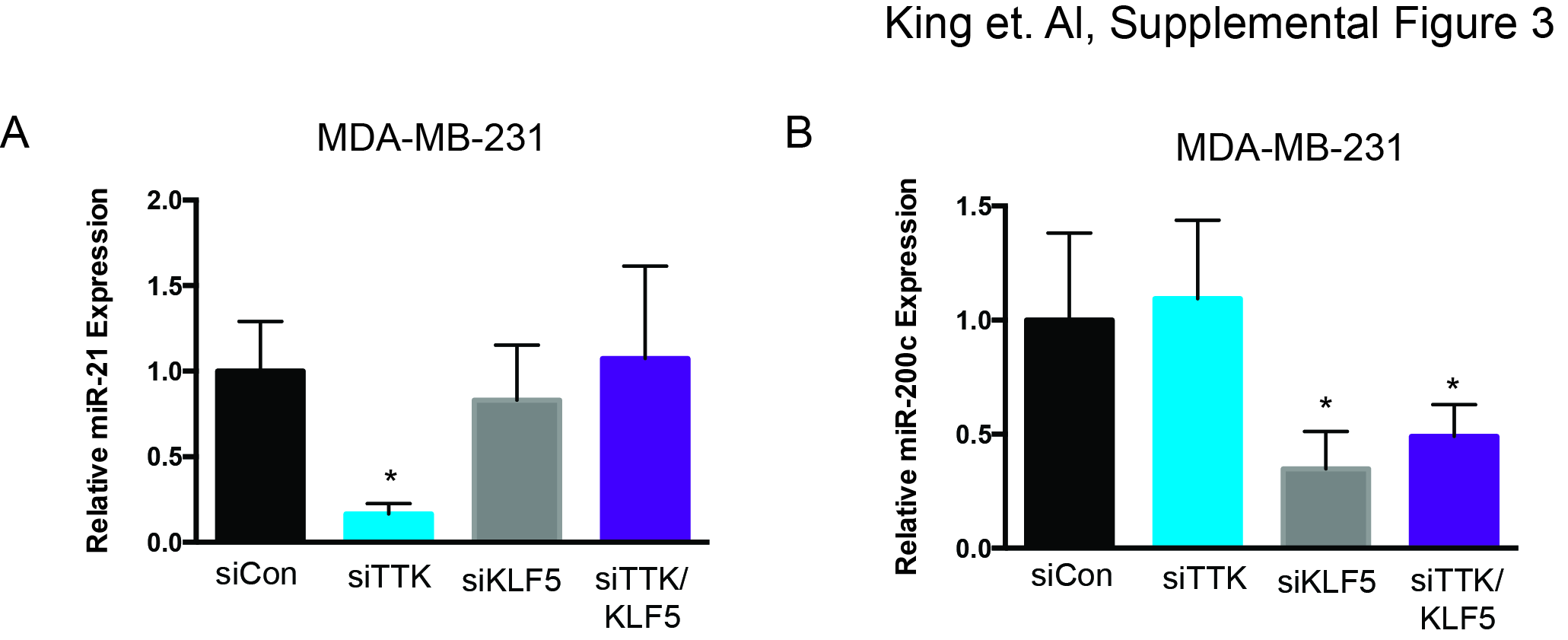

Supplement: Supplementary file 4 — Supplemental Figure 3 [file 41389_2018_77_MOESM4_ESM.tif]

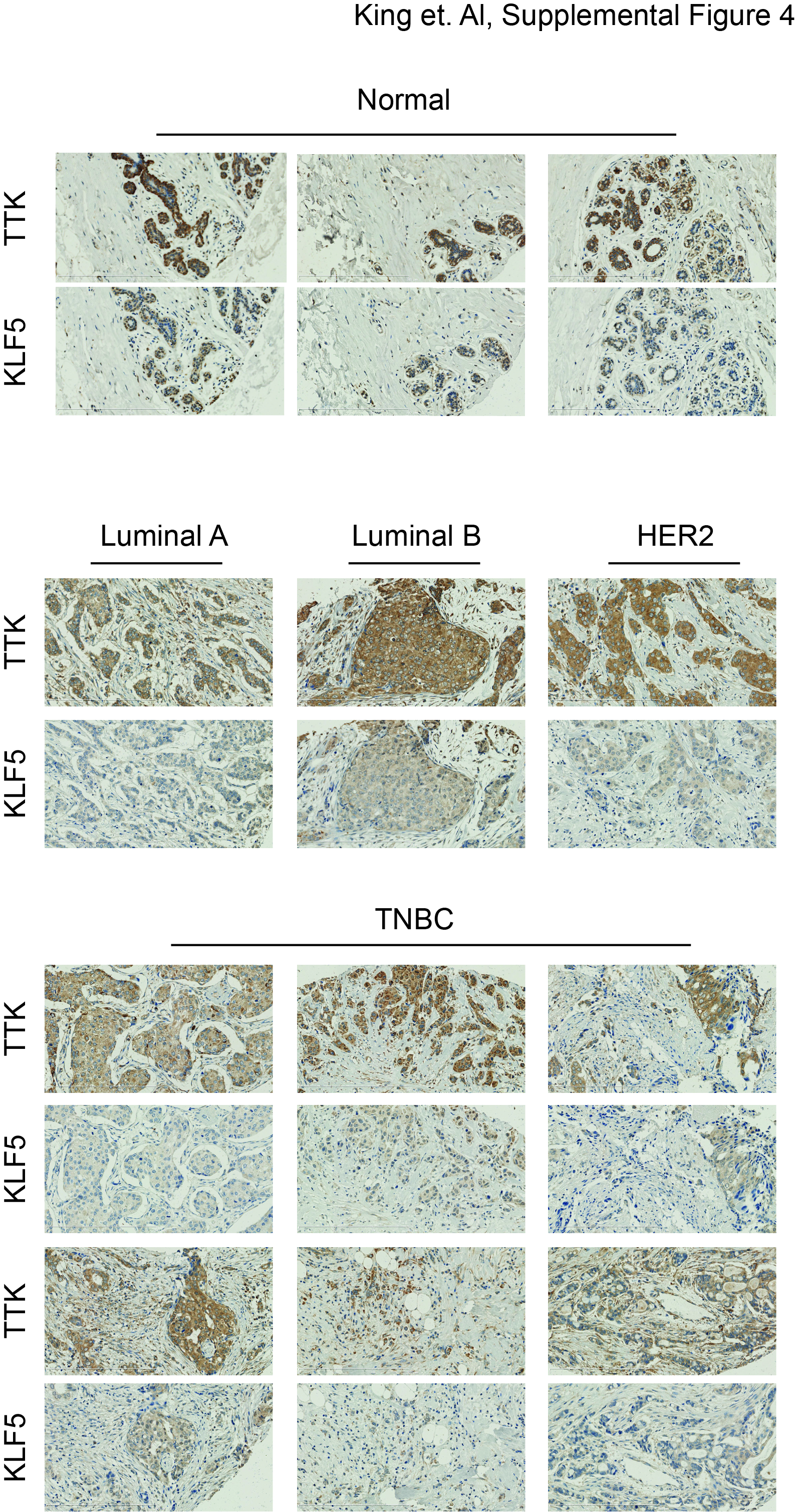

Supplement: Supplementary file 5 — Supplemental Figure 4 [file 41389_2018_77_MOESM5_ESM.tif]
